
JOURNAL INFORMATION
==============================
NLM Title Abbreviation: ScientificWorldJournal
Journal ID: ScientificWorldJournal
NLM Journal ID: 101131163
Journal ID: TSWJ

The Scientific World Journal

ISSN: 2356-6140
EISSN: 1537-744X
Publisher: Wiley

ARTICLE INFORMATION
==============================
PMCID: PMC6084718
PMID: 30147514
DOI: 10.1100/tsw.2001.186
Article ID: 609827
Article version: 1
Subjects: Short Report

Light-induced Photoreceptor Apoptosis in vivo is Caspase Independent and Mediated by Nitric Oxide

Donovan Maryanne *
Carmody Ruaidhri J.
Cotter Thomas G.
Tumour Biology Laboratory, Department of Biochemistry, Lee Maltings, University College Cork, Cork, Ireland
*Maryanne Donovan: madonovan@ucc.ie
Publication date: 2001
Electronic publication date: 2001 Apr 4
Volume: 1
First page: 52
Last page: 52
Copyright: Copyright © 2001 Maryanne Donovan et al.
Copyright year: 2001
License: This is an open access article distributed under the Creative Commons Attribution License, which permits unrestricted use, distribution, and reproduction in any medium, provided the original work is properly cited.
License URL: https://creativecommons.org/licenses/by/3.0/

==============================
Miami *Nature Biotechnology* Short Reports
*TheScientificWorld* (2001) 1 (S3), 52SR
ISSN 1532-2246; DOI 10.1100/tsw.2001.186

### LIGHT-INDUCED PHOTORECEPTOR APOPTOSIS IN VIVO IS CASPASE INDEPENDENT AND MEDIATED BY NITRIC OXIDE

Maryanne Donovan*, Ruaidhri J. Carmody, and Thomas G. Cotter

Tumour Biology Laboratory, Department of Biochemistry, Lee Maltings, University College Cork, Cork, Ireland.
* madonovan@ucc.ie

**INTRODUCTION.** Retinitis pigmentosa (RP) is a genetically diverse disease, characterized by the progressive degeneration of rod and cone photoreceptors. Photoreceptor loss in RP occurs via apoptosis, a mode of programmed cell death, which allows a cell to partake in its own demise. Apoptosis therefore represents a common final pathway in the pathology of RP. However, the molecular events regulating photoreceptor apoptosis in human cases and animal models of RP remain undefined. In this study, we delineate the molecular events occurring during photoreceptor apoptosis in the light-induced retinal injury model of RP.

**METHODS.** *Retinal light damage:* Adult male balb-c mice were exposed to 2 hours of cool white fluorescent light at a luminescence level of 5000 lux. *Intraperitoneal injections:* Mice were injected intraperitoneally with 100mgkg$^{-1}$ of N$^{G}$-nitro-L-arginine methyl ester (L-NAME) in phosphate buffered saline (PBS), or D-NAME (an inactive isomer) in PBS as a control, 1hr before light exposure. Flow cytometry was employed to monitor intracellular calcium levels, generation of reactive oxygen species and mitochondrial membrane depolarisation in untreated animals and in those treated with L-NAME. Caspase-3 activation was assessed by western blot and with the use of the synthetic substrate AcDEVD-pNA. The possible involvement of other caspases was assessed by treatment of the animals with ZVAD.fmk subretinally.

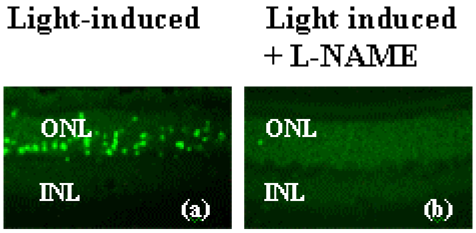

**Detection of DNA strand breaks in photoreceptor nuclei by *in situ* end-labeling (TUNEL).** (a) Retina of mouse sacrificed 14 hours after light exposure show significant labeling of photoreceptors. (b) Retina of mouse treated with 100mgkg$^{-1}$ of the NOS inhibitor L-NAME, 1 hour prior to light exposure. Photoreceptors show no labeling. ONL: Outer nuclear layer, INL: Inner nuclear layer.

**RESULTS.** In this study, we delineate the molecular events occurring during photoreceptor apoptosis in the light-induced retinal injury model of RP. We demonstrate an early increase in intracellular calcium levels during photoreceptor apoptosis, an event that is accompanied by significant superoxide generation and mitochondrial membrane depolarisation. Furthermore, we show that inhibition of nitric oxide synthease (NOS) is sufficient to prevent all features of apoptosis monitored, implicating a key role for nitric oxide (NO) in light-induced retinal degeneration. In addition, our results demonstrate that caspase-3, which is considered to be one of the major downstream caspases of apoptosis, is not activated in this model.

**DISCUSSION.** It has been suggested that inhibition of apoptosis is sufficient for the prevention of retinal degeneration and that by blocking the death of rod photoreceptors and the subsequent death of the cones, visual function may be retained. The data presented here demonstrates that both oxidative stress and elevated calcium contribute significantly to an apoptotic process, which does not involve caspase-3. These findings suggest that therapeutic strategies, which target the initiating signals of apoptosis such as antioxidants or calcium channel blockers, may be of more therapeutic benefit, than caspase inhibitors.

**ACKNOWLEDGEMENTS.** This work was funded by Bausch and Lomb, Ireland and RP Fighting Blindness Ireland.

**REFERENCES.**

1. Heckenlively, J.R. (1988) Retinities Pigmentosa, J.B. Lippincott, Philadelphia, PA
2. Portera-Alilliau, C., Sung, C.H., Nathans, J., and Adler, R. (1994) Proc. Natl. Acad. Sci. U.S.A. 91, 974-978
